# It sounds like a good handover but can I trust it: the correlation between perceived quality and accuracy?

**DOI:** 10.15694/mep.2021.000102.1

**Published:** 2021-04-28

**Authors:** Malcolm Moore, Suzanne Bain-Donohue, Molly Barry, Phillip Gray

**Affiliations:** 1 Australian National University Medical School; 2 Australian National University Medical School

**Keywords:** Clinical handover, work-based assessment, entrustable professional activities, clinical skills, communication skills

## Abstract

This article was migrated. The article was marked as recommended.

Background

Safe handover is crucial in healthcare and is taught in undergraduate and pre-vocational training curricula. It is now considered an Entrustable Professional Activity (EPA). Handover assessment tools have been developed but the correlation between the perceived quality of a handover and its accuracy has not been studied.

Aims

This paper aims to determine the correlation between the perceived quality and the accuracy and safety of handover.

Methods

This descriptive, quantitative study looked at medical students on long-term rural clinical placements who gave clinical handovers to supervisors. The supervisors scored the handovers using the Clinical Handover Assessment Tool (CHAT) and assessed the accuracy and safety of the handover, after seeing the patient. The correlation between handover scores, accuracy and safety was calculated using Cramer’s V coefficient.

Results

114 handovers from 25 students were assessed. The correlation coefficient for a global assessment of quality and accuracy was 0.585 and for safety was 0.583, considered large effects (>0.35). This also held using a checklist quality assessment but less strongly: 0.419, 0.363 respectively.

Conclusion

These findings suggest that handovers that sound ‘good’ are likely to be accurate: clinicians can ‘trust their gut-feeling’. A high quality handover reflects more than the trainee’. clinical reasoning, communication and organisational skills: it suggests that they can provide accurate and safe handover. This supports the use of global assessments of handover as an important part of the multi-source feedback required for summative entrustment decision-making.

## Introduction

### The importance of safe handover

Clinical handover is increasingly important in all areas of healthcare. In hospitals, safer working hours have led to increased transitions of care between shifts ( Schumacher *et al.,* 2012). As patients transition through community care to hospital and back again there are many opportunities for errors in information transfer ( Kripalani *et al.,* 2007; Vermeir *et al.,* 2015).

Recognition of the importance of safe handover - an Entrustable Professional Activity (EPA) - is reflected now in many undergraduate and pre-vocational training curricula ( AAMC, 2014; Liston *et al.,* 2014). Increasingly, handover training of students and trainees uses simulated learning or occurs in the workplace. Various handover frameworks, using acronyms including ISBAR and I-PASS, are taught across jurisdictions and specialties ( WHO, 2011; Feraco *et al.,* 2016). The diversity of handover frameworks reflects the differences in handover required by different specialties and institutions ( Davis *et al.,* 2017). Handovers at a change of shift emphasise the transfer of important data and outstanding tasks. Handover of newly assessed cases in acute settings such as emergency departments, wards and community practice has a greater requirement for competent case description, interpretation and planning. In all of these settings, the accuracy of the handover is crucial. It is important to note, however, that handover is not a simple transfer of information but requires two-way communication to construct an agreed understanding of the patient ( Cohen, Hilligoss and Amaral, 2012).

### Handover assessment

There are various tools to assess and give feedback on handover ( Davis *et al.,* 2017). These tools assess various combinations of handover content, process, organisation and professionalism. Much handover assessment has occurred in a training or research setting where assessment is based on communication of pieces of information from an agreed list ( Abraham *et al.,* 2012; Bates *et al.,* 2014; Thompson *et al.,* 2011). In a simulated learning environment, handover quality and accuracy can be measured against known patient details ( Cunningham *et al.,* 2012; Marshall, Harrison and Flanagan, 2009). In the workplace, receiving clinicians make judgements about quality but can only assess accuracy against a subsequent assessment of the patient. A handover might be given in an acceptable format, with confidence and apparent high quality, but the information might be inaccurate and the reasoning and recommendations faulty or unsafe. There have been no workplace studies of the correlation between the perceived quality of a handover and its accuracy and safety.

### Entrustment decisions

Supervisors are faced with a complex decision when deciding how much to trust handovers they receive and the trainees who are delivering them. The perceived quality of the handover is part of that decision. The complexity of the concept of trust has been described alongside the development of EPAs ( Duijn *et al.,* 2018; Ten Cate, 2017; Ten Cate *et al.,* 2016). Damodaran, Shulruf and Jones (2017) assert that trust in healthcare supervision requires the acceptance of vulnerability and risk. This is particularly true in the process of accepting a clinical handover about a patient who the supervisor might not have seen nor have the means to immediately access.

Ten Cate *et al.* (2016) place entrustment decisions into two categories: *ad hoc* and summative. *Ad hoc* entrustment decisions take trainee factors into account but also consider the supervisor and the context, such as time of day, staffing levels and urgency. Summative decisions focus on the characteristics of the trainee and are much less influenced by the supervisor and the context. These decisions will occur, for example, at the end of training. The authors note the importance of effective assessment and of making ‘safe, effective patient-centred care [as] the frame of reference’ for reaching entrustment decisions. In handover this requires the creation of an accurate picture of the patient’. condition and care needs.

Ten Cate’. work (2017) on entrustment summarises the important factors in decision-making across four variables: the perceived trustworthiness of the trainee; the perceived risks and benefits of entrusting; and the propensity of the supervisor to trust. Perceived trustworthiness is also conceptualised across four variables: ability, integrity, reliability, and humility. Humility includes the trainee’. propensity to acknowledge their limitations and seek help. Duijn *et al.* (2018) added ‘adequate exposure’ to these variables. Ten Cate notes that this model is most suitable for *ad hoc* decisions because summative decisions require a more systematic risk analysis from multiple sources.

There is a diverse range of EPAs and each will require a different method of assessment. In all cases the ‘quality’ of the EPA may not correlate with the ‘perceived trustworthiness’ of a trainee. The assessment can be relatively straightforward when the EPA involves a clearly defined activity. For example, ‘entering prescription orders’ and ‘obtaining informed consent’. two of the Association of American Medical Colleges (AAMC)’. list of 13 EPAs, have outcomes which are relatively clear. More complex activities, such as handover, have less clearly defined outcomes. An impression of a handover’. ‘quality’ might be based on an assessment of the trainee’. apparent competence, communication skill and willingness to ask for help. Whilst these are important factors, they don’. inform the supervisor’. assessment of whether the handover is accurate and safe. This can only be determined by knowing the patient’. ‘real’ condition - information that is often not immediately available to supervisors in the workplace. Supervisors are left to trust the handover based on prior knowledge of the trainee, the handover’. organisation, the confidence of the trainee or their communication skills. We know that a confident, well-organised, affable salesperson can sell an ineffective product.

### CHAT

The Clinical Handover Assessment Tool (CHAT), used in the current study, was developed to enable work-based assessment and feedback; its development and piloting have been described elsewhere ( Moore *et al.,* 2017; Moore and Roberts, 2019). It contains checklist items, based on the elements of ISBAR, providing an objective measure of aspects of ‘quality’. A global rating is based on the assessor’. confidence in the accuracy of handover: ‘How confident am I that I received an accurate picture of the patient?’. In determining this rating the assessor is likely to draw on elements described by ten Cate, above. The CHAT has been used in several sites to assist clinicians to provide feedback to medical students.

### Study setting

The Australian National University (ANU) Medical School’. Rural Clinical School places students in rural locations in the south-east and central west of New South Wales for the third year of their four-year course. They are supervised by clinicians in a mix of community and hospital practice. Many supervising GPs also work in the hospital emergency department and on the wards. Students join the healthcare team and give handover to their supervisors when they have performed an initial assessment of patients. They are required to be assessed on at least ten handovers during the year - five early and five late in the year, using the CHAT, as an opportunity for feedback and learning. All students receive handover training before commencing their placements and are encouraged to use the ISBAR framework in community and hospital.

### Study objective

This study addresses the identified gap in the literature concerning perceived quality by asking, ‘What is the correlation between a handover’. perceived quality and its accuracy and safety?’ This question is important to clinicians who are deciding how much to trust the handover they are receiving. It will also assist supervisors and educators making entrustment decisions in the workplace, highlighting the issue of patient safety.

Whilst the main focus of the study is on accuracy, the issue of patient safety is considered paramount in handover. This issue has been the main driver of efforts to improve healthcare communication ( WHO, 2007). We also consider that accuracy and safety are independent issues and likely to be considered separately by supervisors. We hypothesised that a student seeing a sick patient might score poorly on handover accuracy - ‘ability’ in ten Cate *et al*’. (2016) model but could still recognise the need to safely escalate the patient and seek assistance (‘humility’).

## Methods

This is a descriptive, quantitative study. A cohort of medical students at the end of their long-term rural training was assessed by clinical supervisors on at least five handovers, using the CHAT, followed by feedback and discussion. After subsequently assessing the patient, the supervisors were asked to rate the handover’. accuracy and safety. Two questions were added to the CHAT, the expanded study tool being named CHAT-A. (Appendix 1) The first question concerns accuracy: ‘How closely did the patient’. condition match the picture I received in the handover?’.he second question asks, ‘Were there any issues of patient safety arising from this handover?’.

Global assessment, accuracy and safety were all scored 0,1,2 or 3. Scores were analysed to identify the correlation between CHAT scores and handover accuracy and safety. The Cramer’. V correlation coefficient was selected for use because the small number of scores (in this case 1,2,3) required the data to be treated as categorical ( Mangafiaco, 2016). Checklist scores were aggregated into three categories (≤14, 15-16, 17-18) to facilitate comparison, having the same numbers of rows and columns for crosstab analysis as the global assessment. The value of Cramer’. V is from 0 to 1, where 1 is a perfect association. A value ≥ 0.35 is considered a large effect for a minimum of three rows or columns. This study was approved by the Human Research Ethics Committee at the Australian National University.

## Results/Analysis

Handovers were assessed in five rural towns by 42 clinical supervisors who each completed between 1 and 10 assessments. These occurred in four settings. ( Table 1)

**Table 1:** Site of handover

Site	Student No.
General practice	40
Emergency department	64
Paediatric ward	2
Medical ward	1

25 students (16 female, 9 male) gave 127 handovers - 23 students completed five handovers, two students completed six.

114 handovers without missing data were analysed with 13 handovers excluded as they lacked accuracy and safety scores.

The complexity of handover cases was rated by assessors as low (N=23), medium (N=54) or high (N=22) (15 unrated).

The 114 global assessment (GA) scores were 1 (N=2), 2 (N=45), 3 (N=67). ( Table 2) Of the 45 GA scores of 2, accuracy scores were 2 (N=18) and 3 (N=27). Of the 67 GA scores of 3, accuracy scores were 2 (N=4) and 3 (N=63). In total, 90/114 handovers scored 3 for accuracy. The Cramer’. V correlation coefficient for global score and accuracy was 0.585, p=0.00.

**Table 2:** Global assessment and Accuracy Cross-tabulation

		Accuracy			
		1	2	3	Total
**Global assessment**	**1**	1	1	0	2
	**2**	0	18	27	45
	**3**	0	4	63	67
	**Total**	1	23	90	114

The 114 checklist scores (/18) were: ≤14 (N=14), 15-16 (N=29), 17-18 (N=71). Of the checklist scores of 17-18, accuracy scores were 2 (N=5) and 3 (N=66). ( Table 3) The Cramer’. V correlation coefficient for checklist and accuracy was 0.419, p=0.00.

**Table 3:** Checklist and Accuracy Cross-tabulation

		Accuracy			
		1	2	3	Total
**Checklist grouped**	**≤14**	1	10	3	14
	**15-16**	0	8	21	29
	**17-18**	0	5	66	71
	**Total**	1	23	90	114

The analysis for safety produced similar findings. Cramer’. V for global assessment was 0.583 and for checklist was 0.363, both are considered a large effect although this was close to the cut-off (≤0.35) for the checklist.

The correlation between assessments of accuracy and safety was calculated. ( Table 4) Cramer’. V showed a large effect at 0.817, p=0.00. Of the 23 handovers scoring 2 for accuracy, 11 scored 3 for safety. Of the 90 handovers scoring 3 for accuracy, 3 scored 2 for safety.

**Table 4:** Accuracy and Safety Cross-tabulation

		Safety			
		1	2	3	Total
**Accuracy**	**1**	1	0	0	1
	**2**	0	12	11	23
	**3**	0	3	87	90
	**Total**	1	15	98	114

There was a strong correlation between the GA and the checklist scores ( Table 5): Cramer’. V was 0.459, p=0.00.

**Table 5:** Global assessment and Checklist Cross-tabulation

		Checklist grouped			
		≤14	15-16	17-18	Total
**Global assessment**	**1**	2	0	0	2
	**2**	10	21	14	45
	**3**	2	8	57	67
	**Total**	14	29	71	114

## Discussion

The correlation between global assessment and accuracy in this study (Cramer’. V=0.585) is a large effect (Cramer’. V>0.35). This correlation between the perceived quality and accuracy of handovers gives confidence that ‘good’ handovers can be trusted. This is an important finding for receivers of handover who have no objective evidence for accuracy at the time of handover. These results encourage clinicians to have confidence to trust their ‘gut feeling’ in making *ad hoc* entrustment decisions.

Global assessment showed a stronger correlation with accuracy than checklist although both were strong: Cramer’. V was 0.585 for GA, 0.419 for checklist. Given that handover accuracy is of paramount importance, this study provides support for the use of a global assessment. This difference between GA and checklist is consistent with the previous CHAT study which found differences in the generalisability of checklist and GA scores: GA score outperformed the checklist as a measure of a student’. ability while checklist outperformed GA score on individual handover quality ( Moore *et al.,* 2017). This suggested that the assessor making a global assessment was making an assessment that was broader than scoring the individual handover. If an assessor knows the trainee from experience as a clinical supervisor, the GA provides a means by which they can access that broader experience: this will affect their confidence in the handover. These findings support the use of global assessments in making entrustment decisions because they allow the assessor to draw on the variety of factors identified in the entrustment literature. The validity of global scoring has been debated for decades but there is strong support for its role in grading complex tasks, handover being a good example ( Regehr *et al.,*1998; Sadler, 2009). This approach also aligns with the principles of work-based assessment identified by Crossley and Jolly (2012): notably, the importance of seeking ‘judgements’ rather than focussing on objective observations.

Assessors had misgivings or identified deficiencies in handover quality that weren’. always reflected in the accuracy they determined after seeing the patient. There are several likely explanations for scores with a discrepancy between quality and accuracy. These include a student’. lack of confidence, poor organisation or poor communication skills. Most handovers that scored highly on the GA (3) and checklist (17-18) scored 3 for accuracy: 94% (63/67) and 92.9% (66/71) respectively. However, many handovers were scored as not inspiring full confidence (GA <3) or as deficient (checklist <17). Over half of these then scored 3 for accuracy. 57.4% (27/47) of GAs; 55.8% (24/43) for the checklist.

The correlation between GA and checklist was strong but there were interesting differences between the scores that shed light on the process of assessment. 10 of the 67 handovers with low checklist scores received high GA scores. This is likely to reflect presentations that were initially poor but were able to inspire confidence after significant further interrogation: the checklist rubric is based on the amount of questioning required. Some may reflect poor presentations that had enough clinical evidence or sound reasoning to convince the supervisor. Conversely, 45 handovers receiving a GA of 2 received a range of scores for checklist: ≤14 (N=10), 15-16 (N=21), 17-18 (N=14). Assessors based their lack of confidence on factors beyond the measures of ‘quality’ determined by the checklist rubric.

There was a strong correlation between scores for accuracy and safety (Cramer’. V=0.817) but assessors showed discrimination between these two dimensions. If handovers were accurate they were almost always safe. Handovers that were less accurate could also be seen as safe. The GA was a better predictor of handover safety (Cramer’. V=0.583) than the checklist (Cramer’. V=0.363): this was a larger difference than for accuracy. Again, this is likely to reflect the broader range of factors contributing to the GA. In particular, the student’. ‘humility’ - their propensity to admit limitations and seek help - is a potential strong contributor to an assessor’. estimation of safety. In the current study, most of the supervisors had prior knowledge of the students and some evidence on which to base this assessment.

In this study, the GA performed more strongly than the checklist as a predictor of accuracy and safety. This is an important finding, given the complexity of handover as an EPA ( ten Cate and Young, 2012) and the 21 decision variables identified as relevant to entrustment decision-making ( Duijn *et al.,* 2018). Assessing this complexity is made more difficult because of the nature of handover as a two-way communication ( Cohen, Hilligoss and Amaral, 2012). It is important that assessments determining entrustment decisions take as many of these factors into account as possible. The current results support the use of an appropriate global rating for this assessment, one that aligns with the purpose of handover: to enable an accurate and safe transfer of clinical responsibility. The global assessment used in this study has this alignment.

In this understanding of handover assessment, the role of checklists is probably most valuable in the provision of feedback and training. The experience using the CHAT has shown that a short checklist is extremely useful for work-based training ( Moore and Roberts, 2019). They will also continue to have an important role in providing more detailed information on trainee performance and in contributing to the quality improvement and standardisation of assessments ( Pell, Homer and Fuller, 2015).

This study examined medical students who might be expected to present more inaccurate handovers. However, students in this study scored highly on global assessment and accuracy. This is despite only 23% of cases being rated as low complexity. Students had spent a year on rural placement, receiving frequent handover feedback. The results provide further evidence that students can contribute safely as part of a care team.

### Limitations

Not all supervisors were able to receive training in the use of the CHAT and this might have caused inaccuracies in scoring. However, there were no issues identified in its use.

We were unable, unfortunately, to gather data for early and late stages of placement as planned, due to the COVID-19 pandemic disruption.

It is possible that some assessors adjusted their marking to account for their expectations of a medical student’. level of proficiency. This is not the intention of the checklist rubric. While some assessors may have marked leniently, they made discriminating assessments of accuracy. More study in higher acuity and less trained participants will clarify the findings.

## Conclusion

Handovers that sound good are likely to be accurate. These findings provide evidence for the validity of work-based assessment of handover quality as a measure of a trainee’. ability. A supervisor can be reasonably confident that a high scoring handover reflects more than the trainee’. clinical reasoning, communication and organisational skills. It suggests that they can provide accurate and safe handover. These assessments will be an important part of the multi-source feedback required for summative entrustment decision-making.

## Take Home Messages

• Clinicians receiving handover can usually ‘trust their gut’ when deciding whether to trust a handover’. accuracy and safety
• Global assessment of handover outperformed checklist assessment when predicting accuracy and safety: it is a crucial part of handover assessment
• Handovers that are inaccurate may still be safe providing the presenter conveys the appropriate clinical priorities
• Medical students can give handover safely as part of a care team

## Notes On Contributors

**Malcolm Moore**, MBBS FRACGP MIH, is an academic clinician in the Rural Clinical School at the Australian National University Medical School. He has worked in rural and remote settings and has a research interest in medical communication. ORCID ID: https://orcid.org/0000-0002-1847-6571

**Suzanne Bain-Donohue**, VCHAM, is a Research Officer in the Rural Clinical School at the Australian National University and Manager of Health ANSWERS. Her research interests include rural medical workforce recruitment and retention and medical education.

**Molly Barry**, BBiomedSc, is a medical student at the Australian National University, Canberra.

**Phillip Gray**, MBBS FRACGP, is an academic clinician working in community and hospital settings. He teaches and conducts research through the Rural Clinical School, Australian National University Medical School.

## Figures and Tables

**Table:** 

*PLEASE TICK APPROPRIATE JUDGMENT*	*Not performed competently*	*Able to perform under firm direction*	*Able to perform under modest direction*	*Able to perform under minimal direction*
**Identifies self and patient**	*Provides inadequate details of self and patient despite prompting*	*Provides appropriate details of self and patient after extended prompting*	*Provides appropriate details of self and patient after a small amount of prompting*	*Provides complete details of self and patient before proceeding*
**Identifies main problem**	*Unable to identify the main problems*	*Identifies and prioritises the main problems after extended prompting*	*Identifies and prioritises the main problems with a few further questions being needed*	*Identifies and prioritises the main problems*
**Gives appropriate history**	*The history is unstructured, or contains significant extraneous information.*	*The key features of the history are elicited but frequent clarification by questioning is required*	*The key features of the history are elicited with only a few further questions being required*	*A comprehensive focused history is delivered*
**Gives appropriate examination/ Observations**	*The examination is omitted, or significant parameters suggested by the problem and history are missing*	*The key features of the examination are reported but frequent clarification by questioning is required*	*The key features of the examination are reported with only a few further questions being required*	*A focused examination is reported*
**Makes logical assessment**	*No credible assessment given*	*The assessment correlates the problem, history and examination, and the context of the encounter after much questioning*	*The assessment correlates the problem, history and examination, and the context of the encounter after minimal questioning*	*The assessment fully correlates the problem, history and examination, and the context of the encounter*
**Makes a clear recommendation**	*No credible recommendation is made*	*A credible recommendation is agreed after extended questioning by the receiver.*	*A credible recommendation is agreed after a few further questions by the receiver*	*A credible recommendation is provided*
**Global Rating How confident am I that I received an accurate picture of the patient?**	*Not at all confident*	*I am confident but required extended questioning on several aspects*	*I am confident but required some further questioning*	*I am confident and required little or no questioning*
**Ungraded observation of additional factors impacting the quality of the handover**				
***Please turn over and score the next items AFTER completing the handover assessment and seeing the patient***			

**Table:** 

**How closely did the patient’. condition match the picture I received in the handover?**	*Not at all closely*	*It was inaccurate in some important elements*	*It was mostly accurate*	*I received an accurate picture*
**Were there any issues of patient safety arising from this handover?**	*The handover was unsafe*	*There were several issues of patient safety*	*The handover was mostly safe*	*There were no issues of patient safety*
**Ungraded observation of factors impacting the accuracy or safety of the handover**				

## Appendices

### Appendix 1

Part 1: Clinical Handover Assessment Tool - Accuracy (C.H.A.T. - A)

Details to be completed by Assessor: Date _____________________ Assessor ________________________________

Person giving handover _____________________________________ Site _________________________________

Designation (eg med student, JMO, RN) _______________________ Complexity of case: low ⎕medium ⎕ high ⎕

Part 2: Accuracy and safety assessment
