# Supplementary material for: It sounds like a good handover but can I trust it: the correlation between perceived quality and accuracy?
Source: MedEdPublish (2016). 2021 Apr 28;10:102. [Version 1] doi: 10.15694/mep.2021.000102.1 (PMC10939514; doi:10.15694/mep.2021.000102.1)
Supplement: Peer review history

# Reviewer report: Reviewer response for version 1

**DOI:** 10.21956/mep.18929.r27067

**Published:** 2021-05-27

**Authors:** Trevor Gibbs

**Affiliations:** 1 AMEE

This review has been migrated. The reviewer awarded 4 stars out of 5 I did enjoy reading this paper which I feel covers an important topic in education, and one that has not been studied too much before. The purpose of the paper was clear and the paper was well written and understandable- although I must admit I struggled with the statistics somewhat.As the authors point out, this is a quality assurance tool for students. As I read the paper and read the conclusions, I was wondering what the predictive validity would be of this tool, will the results transpose to the same handover post graduation, when other pressure- time and workload- come to the fore during handover. I would have enjoyed reading the authors' views on thisI think that this is a useful paper for anyone involved in clinical training

Reviewer Expertise:

NA

No decision status is available

# Reviewer report: Reviewer response for version 1

**DOI:** 10.21956/mep.18929.r27066

**Published:** 2021-05-05

**Authors:** Gurjit Sandhu

This review has been migrated. The reviewer awarded 3 stars out of 5 The authors conducted a descriptive quantitative study with medical students on a rural clinical rotation who provided a clinical handover to their supervisor. Supervisors rated the perceived quality of the handover. After seeing the patient, supervisors scored the handover on accuracy and safety using an assessment tool called CHAT. Based on 114 handovers from 25 students, the authors found perceived quality correlated well with the accuracy and safety of a handover. This suggests that supervisors can trust their perceptions about quality of handovers and supports the inclusion of handovers as part of the multi-source feedback.The writing is clear. The title and abstract are reflective of the content. The manuscript appears to adhere to the journal standards. The ideas in this article would be of interest to both residents and faculty in surgical specialties. Background:• The authors have performed a comprehensive literature search and presented a breadth of details. While the entrustment section is well described as a foundational concept, being more precise and narrow in this section would provide a more streamlined alignment with the scope of this manuscript.• Consider moving the CHAT and Study Setting content into the Methods section.Methods:• The scoring was based on 0,1,2 and 3. Explain to the reader how to make sense of the scale, e.g. “where 0 is… and 3 is…”• Include percentages in the results tables. This will prime the reader for the discussion.• Which CHAT items were contributing most to the supervisor’s quality score? Is there variation with how each item loads?• Was there any association with learner demographics? supervisor demographics? (e.g. gender)Discussion:• The authors state that CHAT training may not have received by all supervisors. Was CHAT training part of the study? If ‘yes,’ add this to the Methods.High quality communication, accuracy with patient information during transitions in care, and clinical reasoning are all integral to the handover event. As such, handovers continue to be an important area of investigation. Thank you for the opportunity to review this manuscript.

Reviewer Expertise:

NA

No decision status is available

# Reviewer report: Reviewer response for version 1

**DOI:** 10.21956/mep.18929.r27068

**Published:** 2021-05-02

**Authors:** Rajaei Sharma

**Affiliations:** 1 College of Medicine and Health, University of Exeter

This review has been migrated. The reviewer awarded 2 stars out of 5 Safety of the handover is a vitally important component of medical education and one that, as the authors highlight, has had strikingly little attention paid to it in the literature. This article is useful for both medical educators looking for ways in which to assess core components of safety and the general reader for interest. The abstract is well written and concise. A little more detail could be added to the results section for increased clarity.The introduction provides a nicely detailed background with good grounding in the literature. The authors state that there have been no workplace studies of correlation, but this is a highly specific assessment of handovers. There may well have been other studies about the handover in the workplace and it may be useful to briefly describe any similar or tangential works that may have been carried out.The methods section is clear but for completeness it would be useful to have more detail about how the students were selected, whether there was any recognition of other influential factors such as the students’ general ability or prior experience and how the assessors were trained/standardized. Re-production has not been made easy. These have been addressed to some extent in the discussion only. The final paragraph of the discussion suggests that students may be expected to underperform, but it is likely that the assessors were taking their relatively low experience into account. Should this be compared to junior doctors to assess variation?The finding that the GA performed more strongly than the checklist is a key outcome and should be highlighted. The limitations are self-evident, but could be a little more detailed with suggestions for further works and variations of the study. The conclusion is a little lacklustre relative to the rest of the paper!Overall this is a good article and evidently highlights a void in the literature. Hopefully it will serve as a springboard for many more projects. It is a useful piece as is – revision with attention paid to the aforementioned areas would make it a great read.

Reviewer Expertise:

NA

No decision status is available
